# RNA sequencing and functional studies of patient-derived cells reveal that neurexin-1 and regulators of this pathway are associated with poor outcomes in Ewing sarcoma

**DOI:** 10.1007/s13402-021-00619-8

**Published:** 2021-08-17

**Authors:** Elizabeth Ann Roundhill, Mariona Chicon-Bosch, Lee Jeys, Michael Parry, Kenneth S Rankin, Alastair Droop, Susan Ann Burchill

**Affiliations:** 1 Children’s Cancer Research Group, Leeds Institute of Medical Research, St. James’s University Hospital, Leeds, LS9 7TF UK; 2 Royal Orthopaedic Hospital NHS Foundation Trust, Bristol Road South, Northfield, Birmingham, B31 2AP UK; 3 Translational and Clinical Research Institute, Paul O’Gorman Building, Framlington Place, Newcastle upon Tyne, NE2 4AD UK; 4 Wellcome Sanger Institute, Hinxton, Cambridgeshire, CB10 1SA UK

**Keywords:** Ewing sarcoma, Ewing sarcoma stem-like cells, Patient-derived cells, Neurexin-1

## Abstract

**Purpose:**

The development of biomarkers and molecularly targeted therapies for patients with Ewing sarcoma (ES) in order to minimise morbidity and improve outcome is urgently needed. Here, we set out to isolate and characterise patient-derived ES primary cell cultures and daughter cancer stem-like cells (CSCs) to identify biomarkers of high-risk disease and candidate therapeutic targets.

**Methods:**

Thirty-two patient-derived primary cultures were established from treatment-naïve tumours and primary ES-CSCs isolated from these cultures using functional methods. By RNA-sequencing we analysed the transcriptome of ES patient-derived cells (*n* = 24) and ES-CSCs (*n* = 11) to identify the most abundant and differentially expressed genes (DEGs). Expression of the top DEG(s) in ES-CSCs compared to ES cells was validated at both RNA and protein levels. The functional and prognostic potential of the most significant gene (neurexin-1) was investigated using knock-down studies and immunohistochemistry of two independent tumour cohorts.

**Results:**

ES-CSCs were isolated from all primary cell cultures, consistent with the premise that ES is a CSC driven cancer. Transcriptional profiling confirmed that these cells were of mesenchymal origin, revealed novel cell surface targets for therapy that regulate cell-extracellular matrix interactions and identified candidate drivers of progression and relapse. High expression of neurexin-1 and low levels of regulators of its activity, APBA1 and NLGN4X, were associated with poor event-free and overall survival rates. Knock-down of neurexin-1 decreased viable cell numbers and spheroid formation.

**Conclusions:**

Genes that regulate extracellular interactions, including neurexin-1, are candidate therapeutic targets in ES. High levels of neurexin-1 at diagnosis are associated with poor outcome and identify patients with localised disease that will relapse. These patients could benefit from more intensive or novel treatment modalities. The prognostic significance of neurexin-1 should be validated independently.

**Supplementary Information:**

The online version contains supplementary material available at 10.1007/s13402-021-00619-8.

## Introduction

Ewing sarcoma (ES) is a tumour of bone and soft tissues, commonly arising in young people aged 10–25 years [1]. Multi-agent systemic chemotherapy, surgery and radiotherapy have improved the outcome for some patients, although less than two-thirds will survive 5 years beyond diagnosis. Twenty-five percent of patients present with metastatic disease, most frequently in multiple sites including bone/bone marrow/lungs or lungs only. These patterns of metastasis are associated with overall survival rates of 10–20% and 40%, respectively [2]. Disease progression occurs in approximately 50% of all patients, usually within two years of diagnosis. Survival after recurrence is just 10–15% [3, 4]. Patients diagnosed with localised ES generally have a better outcome, with 55 to 70% of them achieving 5 year event-free survival [5]. However, 30–40% of patients with localised tumours will develop multi-drug resistant (MDR) disease leading to relapse and poor outcomes typically associated with widespread metastatic disease, with 5 year survival of less than 10% [6]. Identification of patients with localised disease that do badly at diagnosis could mean they are offered more intensive or alternative experimental treatments, which may improve outcomes. For all patients that survive therapy, 1 in 10 will relapse up to 20 years after initial diagnosis [7], many experiencing treatment-induced morbidity [8–10]. Therefore, the development of molecularly targeted therapies to minimise morbidity and improve outcomes for patients with ES are urgently needed.

ES has been described as a cancer stem cell-like driven disease [11, 12], although this is controversial [13]. ES stem-like cells (ES-CSCs) have the ability to both survive chemotherapeutic insult and enable re-population of the tumour at primary and/or secondary metastatic sites by self-renewal (SR), migration and invasion [14]. Therefore, improved outcome for some patients will only be achieved when drugs to eradicate ES-CSCs are introduced into the clinic. We have hypothesised that a combination of drugs to eradicate ES-CSCs with cytotoxic chemotherapy to destroy the tumour bulk will prevent the development of metastatic multi-drug resistant (MDR) disease and improve patient outcomes. This strategy may also minimise treatment-induced toxicity in case the CSC targeted therapy and chemotherapy have synergistic activity, allowing a reduction in the amount of chemotherapy with no loss of activity.

Sarcoma- and ES-CSCs have most frequently been identified by cell surface expression of the glycoprotein prominin-1 (CD133) [11]. In addition, cell surface expression of the tyrosine kinase protein c-Kit (CD117), low affinity nerve growth factor receptor (CD271) and octamer-binding transcription factor 4 (OCT4, [12]) or intracellular aldehyde dehydrogenase (ALDH [15]), the ability to form 3D spheres [16] and the expression and activity of ATP-binding cassette (ABC) transporter proteins [17] have been employed to enrich for CSC populations. These studies have largely used established cell lines, with a single study investigating ES-CSCs among primary patient-derived cells by investigating cell surface expression of CD133 [11]. However, marker-based enrichment methods can fail to robustly isolate the complete CSC population, reflected by CSC characteristics of CD133-negative cells [18]. Moreover, expression of CSC related proteins may fluctuate throughout the cell cycle [19] and be modified by the cellular microenvironment [20], thereby reducing their value.

In this study, we have adopted a functional approach to enrich for ES-CSCs with MDR and SR ability from primary patient-derived ES cells, as we reported previously in osteosarcoma [21]. Since ES, like other gene fusion-driven cancers of young people, have few recurrent mutations [22–24] we used total ribonucleic acid (RNA) sequencing to characterise patient-derived primary ES cells and daughter ES-CSCs. By differential expression analysis [25], we compared the transcriptome of patient-derived parental and daughter MDR/SR progeny to identify candidate drivers of the ES-CSC phenotype. Potential associations with clinical outcome and biological relevance of the most abundant differentially expressed ES-CSC genes were explored to prioritise candidate risk biomarkers and putative therapeutic targets, with the future goal of developing more personalised therapeutic strategies.

## Materials and methods

### Patient samples, primary patient-derived cell cultures, cell lines and clinical data

Treatment-naïve tumours from 116 young people with a confirmed diagnosis of ES between 1998 and 2017 were included, i.e., fresh tumours from which primary cultures were established (*n* = 32, Additional file 5, Table S2), frozen tumours (*n* = 47, Additional file 9, Table S6) and formalin-fixed paraffin-embedded (FFPE) tumours (*n* = 37, Additional file 9, Table S6). Informed consent and ethical approval for the collection of the tumours was obtained through GenoEWING (IRAS 167880, EDGE 79301). Immunohistochemistry (IHC) was performed on frozen or FFPE tumour samples from the Children’s Cancer and Leukaemia Group Tissue Bank (MREC 98/4/023; biological study 2010 BS03) or the Newcastle Biobank (LREC 17/ND/0361, IRAS 233551). The minimum period of follow up for all patients was 24 months (730 days). Patients received risk-adapted neoadjuvant and adjuvant chemotherapy with surgery, with or without radiotherapy, to control primary and possibly metastatic disease [26]. Cell lines were used as positive controls (Additional file 1, Data S1). The results and methods described in this study are based on the Reporting Recommendation for Tumour Marker Prognostic Studies (REMARK) guidelines [27].

Fresh tumours (*n* = 32) were obtained from patients undergoing surgery at the Royal Orthopaedic Hospital, Birmingham between May 2015 and March 2018. Samples were collected in 15 ml Leeds Antibiotic Media and transported at room temperature to Leeds, where they were processed immediately as previously described [21]. Cells attached to bone were removed using trypsin:ethylenediaminetetraacetic acid (EDTA; 1:1; trypsin 0.25% and EDTA 0.1% both *w*/*v* in phosphate buffered saline (PBS)) at 37 °C for 30 min.

The prognostic potential of candidate drivers of ES-CSCs was explored by IHC of tumours collected at diagnosis. The potential association with clinical outcome was first explored by IHC of frozen tumour samples mounted in Optimum Cutting Temperature compound (OCT; Merck Biosciences) from 47 patients (cohort 1). Tumours were collected between 1998 and 2006 from patients with a median age of 12 years, range 5–20 years. The median follow up time and time to a first event was 912 and 501 days, respectively; 57% of patients had an adverse event. The prognostic potential of the most significant differentially expressed candidate driver gene (neurexin-1) was assessed in a second cohort of 37 FFPE tumours (cohort 2), collected between 2001 and 2017. The median age of ES patients at diagnosis in cohort 2 was 13 years, range 2–38 years. The median follow up time and time to a first event was 2499 and 1747 days, respectively; 36% of patients had an adverse event.

### ES confirmation of cultured cells

Cytological features of patient-derived primary cells were confirmed by light microscopy (Zeiss Axioplan microscope; Zeiss, UK) of haematoxylin stained cells centrifuged onto slides (1000 g for 3 min; Rotix 32A Hettich Zentrifugen). Fluorescence in situ hybridisation (FISH) using a Vysis EWSR1 Break Apart FISH Probe (3 N5920, Vysis, Abbott Laboratories Ltd., UK) and reverse transcriptase polymerase chain reaction (RT-PCR) for Ewing sarcoma breakpoint region 1 (EWSR1)-erythroblast transformation specific (ETS) fusion transcripts were used to check cells for pathognomonic EWSR1 gene rearrangements. Expression of CD99 antigen (CD99) was confirmed by immunocytochemistry (ICC) of cytospins and Western blotting [28] (Additional file 2, Data S2).

### Phenotypic characterisation of ES cells

#### Self-renewing (SR) ability

A single cell (Poisson distribution probability of λ < 1 = 0.9) was seeded into each well of 10 Primaria™ 96-well plates (Corning) and the numbers of wells containing ≥ 5 cells were recorded after 21 days by light microscopy (Olympus CKX41) [21]. Where possible, SR cell populations were propagated to establish daughter cell cultures.

#### Response to cytotoxics

Patient-derived primary ES cells (1 × 10^3^) and TC-32 (positive control, 6 × 10^3^) cells were seeded in Primaria™ 6-well plates (Corning), allowed to adhere overnight and then treated with doxorubicin or vincristine (1–200 nM). After 72 h the media were replaced, colonies maintained for an additional 7 days, fixed and stained with crystal violet (0.25% (*w*/*v*) in methanol:ddH2O; Sigma-Aldrich) [29]. Colony numbers per well were counted using Quantity One Software (Bio-Rad).

#### Migration

Migration over 72 h was determined as previously described [21]. Migration index (MI) = total migrated area relative to the size of the spheroid core at 0 h.

### Transcriptome analysis using total RNA sequencing and differential expression analysis

Total RNA libraries were prepared from 1 μg RNA with an RNA integrity number > 9 extracted from ES primary patient-derived cultures (*n* = 24) and ES-CSCs (*n* = 11) using a TruSeq Stranded Total Library Preparation kit with Ribo-Zero Human (Illumina®, CA, USA). Four samples were pooled (100 ng per sample) before paired-end sequencing using an Illumina® HiSeq3000 apparatus (151 cycles, Illumina®). FASTQ files were downloaded and reads pre-processed using cutadapt [30]. Briefly, low quality reads and any adapter contamination were removed and post-trimmed reads aligned to Gencode human 38 release 25 (GCh38_25) by 2-pass alignment using Spliced Transcripts Alignment to a Reference ([31]; STAR). Differential expression of RNAs between sample groups was identified using DESeq2 [25, 32]. Adjusted *p* values < 0.001 were considered significant.

The most significant differentially expressed RNAs from the RNA sequencing data were validated using reverse transcriptase quantitative polymerase chain reaction (RT-qPCR) and confirmed at the protein level by ICC of cytospins (Additional file 3, Table S1).  

Gene lists were analyzed using the Search Tool for Retrieval of Interacting Genes/Proteins (STRING) database (http://string-db.org, [33]) to identify any interactions between the genes and their reported biological function(s). Interaction Confidence Scores (ICS) were assigned to each protein association and ranked from 0 to 1, where 0 is least likely to be correct and 1 most likely accurate. An ICS of 0.5 indicates that every second interaction might be a false positive. Therefore, only genes with an ICS of 0.5 or greater were imported into Cytoscape v3.7.1 (cytoscape.org, supported by NRNB and NIHR) for visualization. Genes predicted to be expressed on the cell surface were identified by interrogating an ES surfaceome gene expression database (www.imm.ox.ac.uk/research/units-and-centres/mrc-molecular-haematology-unit/research-groups/rabbitts-group/more-from-the-rabbitts-group/surfaceome-database, [34]). Using this tool RNA species are classified as gold or silver, where a classification of gold represents a protein with known cell surface expression and silver a protein predicted to be expressed at the cell surface [34]. The predicted subcellular localisation of protein products in cancer and normal cells was confirmed by interrogation of the published literature and the Human Protein Atlas database (www.humanproteinatlas.org). The FASTQ files of sequenced ES and ES-CSCs are available in the Research Data Leeds Repository (University of Leeds), Burchill, Susan and Roundhill, Elizabeth (2020): Total RNA sequencing of patient-derived Ewing sarcoma and Ewing sarcoma CSCs University of Leeds [Dataset] 10.5518/887

Following the removal of low-quality reads and adapter contamination, 99 ± 1% (range 92–99%) of reads were retained. Consistent with its role as a major effector of the chromosome X inactivation process in females [35], expression of the X-inactive specific transcript (XIST) was low in male RNA (mean normalised read count = 4 ± 5, range 0–22) and significantly higher in females (mean normalised read count = 8326 ± 6112, range 782–19,545; *p* < 0.0000004). Confirming the pipeline for analysis of genes in the pseudo-autosomal regions of the X and Y chromosomes (Additional file 6, Table S3), there was a positive correlation between the read count of CD99 by RNA sequencing and RNA detected by RT-qPCR (R^2^ = 0.898).

### IHC of candidate genes and regulators of their activity

Expression of the top significant differentially expressed genes (DEGs) was evaluated at the protein level by IHC (Additional file 4, Data S3). The level of expression was semi-quantified using the H-score, assessing both the intensity of staining and the percentage of positive cells [36], independent of clinical outcome data.

### Knock-down of neurexin-1 by shRNA and siRNA

ES cells were infected with neurexin-1 (sc-42,050-V) short hairpin RNA (shRNA) lentiviral particles targeting both neurexin-1α and -β isoforms or scrambled sequence controls (sc-108,080; Santa Cruz Biotechnology, Inc.; each shRNA pool containing 3–5 constructs [37]). Infected cells were selected in puromycin (0.5 μg/μl; Sigma-Aldrich) for 10 days before placing them in normal growth media. Knock-down of protein expression after infection was confirmed by Western blotting and ICC. Viable cell numbers over time (24–72 h) were measured using a trypan blue exclusion assay [21] and proliferation using Cell Trace™ CFSE [38]. In the proliferation assay, cells were labelled with carboxyfluorescein succinimidyl ester (CFSE) (0.5 nM) and harvested at 24–72 h, after which fluorescence was measured using an Attune NxT Flow Cytometer (Thermo Fisher Scientific). The effect of doxorubicin and vincristine treatment for 72 h on colony forming efficiency was examined using a cytotoxicity assay (see Phenotypic characterisation of ES cells, above).

SH-SY-5Y (1 × 10^6^) cells, which express high levels of neurexin-1 and were used as a positive control, were electroporated (X-005, Nucleofector™ 2b Device (Amaxa, Lonza, UK) with an siRNA scrambled control (siControl, 50 nM, sc-36,869, Santa Cruz Biotechnology, Inc) or siRNA targeting neurexin-1 (siNRXN1, 50 nM, sc-42,050, a pool of 3 target-specific 19–25 nt siRNAs, SantaCruz Biotechnology, Inc). Knock-down of RNA expression was confirmed by RT-qPCR. Single cells electroporated with siNRXN1 or siControl were seeded into Ultra-Low Attachment plates (Corning, supplied by VWR International, UK) and 3D spheroid formation imaged by light microscopy (Olympus CKX41) after 7 days. The numbers and sizes of spheroids were quantified using ImageJ (NIH, USA).

### Survival and statistical analyses

Results were linked to clinical outcome data in R (R version 3.4.0). The prognostic value of proteins was evaluated using the Cox proportional hazards regression model, the optimal cut-point in the data being determined using the Harrell’s C index [39]. The Cox model was then performed using the defined cut-point and Kaplan-Meier (KM) plots generated using the Survminer package and ggplot; Cox model confidence intervals are included on each KM plot. The potential association of targets with outcome was compared to each other and with patient age and metastasis at diagnosis [40] using a multivariable, univariate Cox model correcting for multiple observations. Patients with missing clinical data were excluded (*n* = 16). The number of patients included in each KM analysis are given on each plot.

Statistically significant differences were determined using a non-parametric Mann-Whitney two-tailed t-test or analysis of variance (ANOVA) with a Tukey’s post-hoc test. Correlations were determined using a Pearson’s correlation coefficient (r). Non-linear regression analysis was used to evaluate differences in viable cell number, proliferation and response to chemotherapy. Statistical analyses were performed using GraphPad PRISM 7.03 (GraphPad Software, San Diego, USA).

## Results

### Patient-derived ES cell cultures express CD99 and pathognomonic EWSR1-ETS gene fusions

**Fig. 1:**
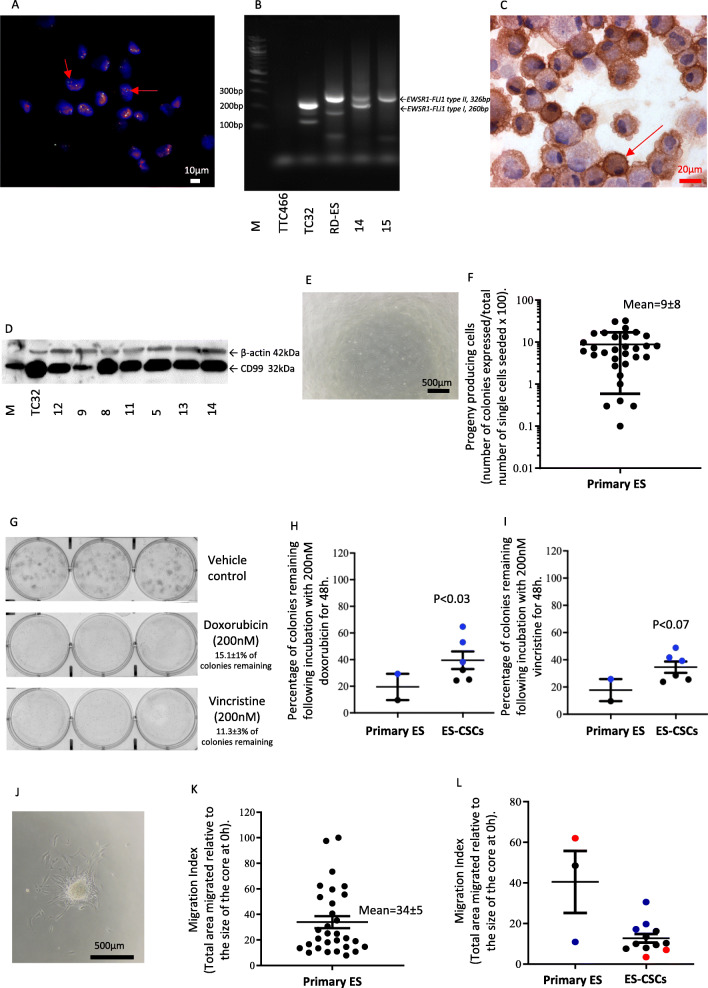
**Characterisation of patient-derived ES and ES-CSC cultures and response to cytotoxics.** (**A**) Image of patient-derived primary ES cells (sample number 32) labelled using a Vysis EWSR1 Break apart FISH Probe. Red arrows = cells containing both a red and green immunofluorescent signal, indicating an EWSR1 gene translocation. (**B**) Representative ethidium bromide stained agarose gel illuminated by UV-light of EWSR1-FLI1 RT-PCR products generated from patient-derived primary ES cultures 14 and 15, confirming both cultures to contain EWSR1-FLI1 products. Positive controls include TC-32 = EWSR1-FLI1 type I (260 base pairs (bp)) and RD-ES = EWSR1-FLI1 type II (326 bp). Negative control = TTC466 which contains an EWSR1-ERG fusion. (**C**) ICC of patient-derived parental culture 27 for CD99. Red arrow = positive CD99 membrane expression. (**D**) Western blot for CD99 protein expression in patient-derived primary ES cell cultures 12, 9, 8, 11, 5, 13 and 14. Equal protein loading was confirmed by probing for β-actin expression. Protein extract from TC-32 cells was included as a positive control. M = molecular weight markers. (**E**) Light microscopy of single cell derived colony 6 established from patient-derived primary ES culture 23; 23.CSC6. (**F**) Percentage of single cell derived clones from patient-derived primary ES cultures. Clones contained >5 cells and are expressed as percentage of the total number of individual cells seeded; results are presented as mean ± SD (*n* = 960 per ES culture). (**G**) Crystal violet staining of primary ES colonies derived from ES culture 14 after treatment with vehicle control, doxorubicin (200 nM) and vincristine (200 nM) for 48 h. Cells were then washed and maintained in appropriate media for an additional 7 days before fixing and staining with crystal violet (0.25%, *w*/*v* methanol:ddH_2_O). Colony numbers were counted using Quantity One Software. Results are shown as mean ± standard error of the mean (SEM) of 3 independent experiments. (**H**) Surviving colonies in paired patient-derived parent primary ES cells (*n* = 2) and ES-CSCs (*n* = 6) following treatment with doxorubicin (200 nM). Results are shown as mean ± SEM of 3 independent experiments. Black = parental culture and matched ES-CSCs from sample 17, blue = parental culture and matched ES-CSCs from sample 23. (**I**) Surviving colonies in matched parent (*n* = 2) and paired ES-CSCs (*n* = 6) following treatment with vincristine (200 nM) Results are shown as mean ± SEM of 3 independent experiments. Black = parental culture and matched ES-CSCs from sample 17, blue = parental culture and matched ES-CSCs from sample 23. (**J**) Light microscopy image of migrating ES cells (patient-derived primary culture 15). (**K**) Migration index of patient-derived primary ES cultures (*n* = 33). Results are shown as mean ± SEM of 3 independent experiments. (**L**) Migration index of ES parent culture (*n* = 3) and paired ES-CSCs (*n* = 11; mean ± SD). Red = parental cultures and matched ES-CSCs from sample 11, black = parental cultures and matched ES-CSCs from sample 17, blue = parental cultures and matched ES-CSCs from sample 23

Propagated patient-derived primary cells had a typical small round cell morphology (69%; 22/32) with scant cytoplasm, round nuclei, smooth distinct membranes and a single nucleolus [41]. Eighteen percent (6/32) were atypical (large irregular nuclei with vesicular chromatin) and 13 % (4/32) intermediate between typical and atypical. All cultures (32/32) contained a translocation involving the EWSR1 gene from chromosome 22q12 detected by FISH and/or RT-PCR (Fig. 1 and Additional file 5, Table S2). Typical of ES, cells were positive for membrane expression of CD99 (Fig. 1C), which was confirmed by Western blotting (Fig. 1D) and RNA sequencing (Additional file 5, Table S2).

### All patient-derived ES cell cultures contain a MDR SR cell population

All patient-derived cultures contained cells that were capable of SR from a single cell (Fig. 1E and F, mean progeny producing cells per culture = 9 ± 8%, range 0.1–31%), consistent with the premise that ES is a CSC driven cancer. Of these single cell-derived cultures, 18 daughter progeny were expanded and propagated for downstream analyses. In agreement with the hypothesis that CSCs evade chemotherapy, the SR ES cells were more resistant to both doxorubicin (200 nM, mean colonies remaining = 44 ± 7% *p* = 0.03) and vincristine (200 nM, mean colonies remaining = 41 ± 8% *p* = 0.07) than the matched parental cells (mean colonies remaining = 20 ± 12% and 17 ± 10%, respectively; Fig. 1G, H, I and Additional file 5, Table S2). Consistent with a MDR phenotype, there was a correlation between the effect of doxorubicin and vincristine (R^2^ = 0.8478; Additional file 5, Table S2). Hereafter these drug resistant daughter cells, with single cell SR ability are referred to as ES-CSCs.

All primary patient-derived ES cell cultures contained migratory cells, with a mean MI of 34 ± 25, range 7.8–100 (Fig. 1J, K and Additional file 5, Table S2). However, there was no statistically significant difference between the MI of patient-derived primary ES cultures and daughter ES-CSCs (12.7 ± 7.3, range 3.42–30.54, *p* = 0.09; Fig. 1L).

### Parental ES and daughter ES-CSCs share transcriptional profiles of mesenchymal stem cells

Parental ES cells and daughter ES-CSCs share a transcriptional fingerprint of embryonic stem cells ([42, 43]; ESCs) and mesenchymal stem cells ([13, 44]; MSCs), consistent with the hypothesis that ES arise in cells of mesoderm origin. The prognostic significance of MSC and ESC associated genes shared by primary ES cells was examined in a publicly available RNA dataset of diagnosis ES tissues (GSE17618, Additional file 6, Table S3). In these data c-KIT predicted event free survival (EFS; Kaplan-Meier (KM) *p* = 0.012, Hazard ratio (HR) = 2.37 and HR associated *p* value = 0.015) and overall survival (OS; KM *p* = 0.039, HR = 2.21 Hazard ratio *p* value (HRp) = 0.044), suggesting that genes of the mesenchymal lineage may be candidate biomarkers of poor outcome. However, in a phase II trial of imatinib mesylate, which inhibits c-KIT, platelet derived growth factor receptors and BCR-ABL, only 1/24 ES patients responded [45] and in a second study of 13 ES patients no responses were observed [43]. The hypothesis that genes of the mesenchymal lineage expressed by ES cells may be candidate risk biomarkers and/or therapeutic targets requires further investigation.

### Identification of ES and ES-CSC specific cell surface proteins as putative biomarkers and candidate targets for therapy

**Fig. 2:**
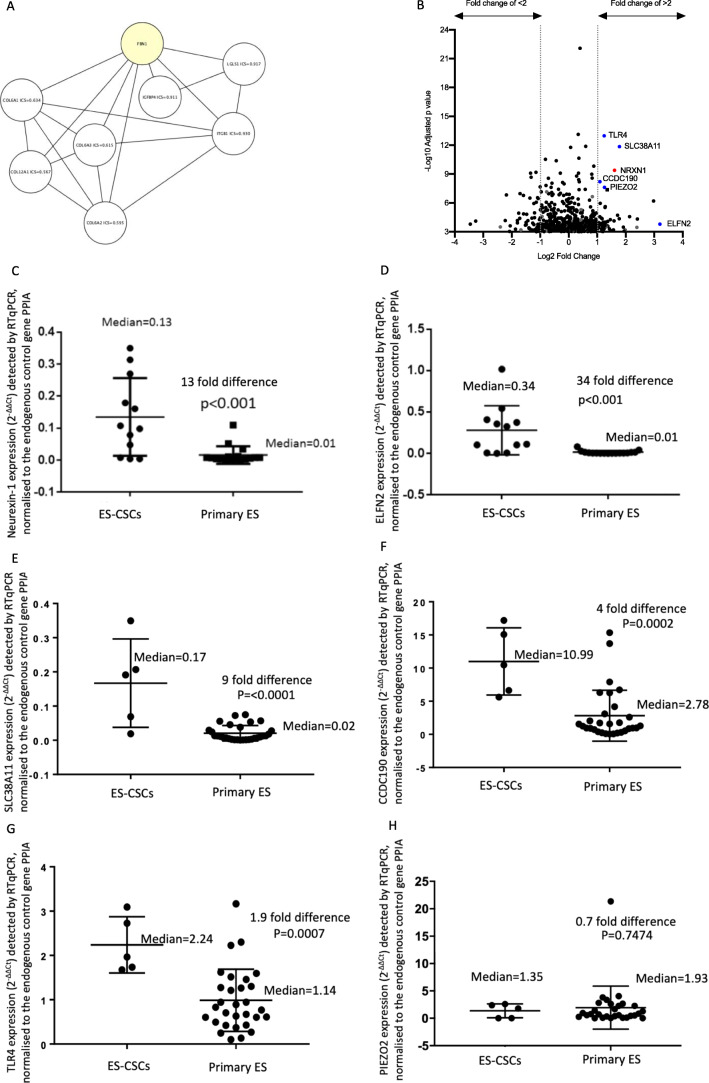
**Highly expressed cell surface proteins and validation of differentially expressed mRNAs between ES and ES-CSCs.** (**A**) Interactions between proteins predicted to have high levels of cell surface expression in patient-derived ES cells and ES-CSCs and low expression in normal tissues (www.humanproteinatlas.org), generated using the protein-protein interaction tool STRING database (http://string-db.org) and visualised using Cytoscape v3.7.1 (www.cytoscape.org). (**B**) Butterfly plot of mRNAs with signficant (adjusted *p* < 0.001) differential expression in patient-derived parental ES and ES-CSCs. Black circle = all differentially expressed genes with an adjusted *p* value < 0.001. Dotted line = 2 fold increase (fold change of > 2) or 2 fold decrease (fold change of < 2) in mRNA level in ES-CSCs compared to ES cell cultures. Target mRNAs identified for validation by RT-qPCR are labelled; blue and red circles. Grey circle = currently unannotated genes. Quantification of target mRNAs using RT-qPCR in patient-derived primary ES and ES-CSCs; (**C**) neurexin-1 (NRXN1), (**D**) ELFN2, (**E**) SLC38A11, (**F**) CCDC190, (**G**) TLR4 and (**H**) PIEZO2. Median gene expression is reported as 2^-ΔΔCt^ (results shown as median ± SD). RNA expression was compared between populations using a non-parametric Mann-Whitney two-tailed t-test

Since cancer cell-surface proteins (the so called cancer cell surfaceome) may represent attractive targets and biomarkers for anti-cancer treatment [34, 46], we interrogated our RNA sequencing data to identify genes predicted to be highly expressed in all ES and ES-CSCs. Genes were ranked on mean read count, localisation at the cell surface (with gold status using the Surfaceome database; [34]) and reported to have low or no expression in normal tissues (expression in < 5 normal tissues; Human Protein Atlas). Analysing our panel of patient-derived primary ES cell cultures and ES-CSCs, we identified 11 genes for further investigation (Table 1). Although no specific gene ontology (GO) pathway terms were represented in this list, 8/11 identified genes have been reported to interact (STRING database, http://string-db.org [33], ICS > 0.5; Fig. 2A), including the top hit FBN1 which is associated with 7/8 partners in the list (Table 1, Fig. 2A), suggesting that these genes are part of the same cellular pathways. Exploring the surfaceome of ES cell lines, 10 cell surface genes with high expression in ES cells compared to MSCs have previously been explored as candidate therapeutic targets [34]. We confirmed expression of 9/10 of these genes in patient-derived primary ES and ES-CSCs, 7 of which we predict will be part of the ES surfaceome (Additional file 7, Table S4).

**Table 1:** Highly expressed cell surface target genes in patient-derived primary ES cells and daughter ES-CSCs

Gene name and protein product		Mean RNA read count in patient-derived primary ES and ES-CSCs, determined by total RNA sequencing.	Rank*	STRING ICS with Fibrillin 1
FBN1	Fibrillin 1	313,166	11	–
COL6A3	Collagen type VI alpha 3 chain	296,145	13	0.615
COL12A1	Collagen type XII alpha 1 chain	295,027	14	0.567
ITGB1	Integrin subunit beta 1	160,061	27	0.930
COL6A2	Collagen type VI alpha 2 chain	138,252	30	0.595
LRP1	LDL receptor related protein 1	111,028	37	NI
CLIC4	Chloride intracellular channel 4	104,677	39	NI
COL6A1	Collagen type VI alpha 1 chain	99,781	45	0.634
LGALS1	Galectin 1	79,551	62	0.917
IGFBP4	Insulin like growth factor binding protein 4	74,656	67	0.911
Serpine2	Serpin family E member 2	73,506	70	NI

*Rank based on mean read count from RNA sequencing. ICS = Interaction Confidence Score identified in STRING; 0 = least likely to be correct and 1 = most likely to be correct. NI = no interaction reported in the STRING database

### Identification of candidate genes driving the ES-CSC phenotype

Comparison of the total RNA profiles of ES and matched ES-CSCs revealed 561 differentially expressed genes (adjusted *p* value < 0.001), representing 1.5% (561/36575) of all genes detected in ES and ES-CSCs. To prioritise genes that are differentially expressed in ES compared to ES-CSCs for further investigation, genes were ranked based on significance (adjusted *p* value) and fold change in expression at the RNA level (from DESeq2 analysis; Burchill, Susan and Roundhill, Elizabeth (2020): DeSeq2 output comparing the total RNA sequencing gene expression data of paired patient-derived primary Ewing sarcoma cultures and Ewing sarcoma CSCs. University of Leeds [Dataset]. 10.5518/886). Of the 561 differentially expressed genes, 9 were identified in the top 100 differentially expressed genes based on both fold change in normalised read-count and adjusted *p* value (Fig. 2B). Expression of 6 of these 9 genes was significantly increased in ES-CSCs compared to parental cultures (Table 2). Since we were seeking genes that are highly differentially expressed and might be used to select patients for targeted treatment, we went on to confirm the expression of these 6 genes at the RNA and protein level and investigate their potential association with clinical outcome.

**Table 2:** High differentially expressed RNAs between patient-derived primary ES cells and ES-CSCs

RNA expression determined by comparison of total RNA sequencing of patient-derived primary ES cells and ES-CSCs(1 = increased, 0 = decreased)	Gene	Fold change in RNA expression comparing normalised total RNA sequencing reads from ES-CSCs and parental cells	Adjusted *p* value
1	ELFN2*	9.2	0.00016249
1	NRXN1*	3	4.07 × 10^−10^
1	SLC38A11*	3.4	1.40 × 10^−12^
1	**PIEZO2**	2.4	2.43 × 10^−8^
1	**TLR4**	2.4	1.06 × 10^−13^
1	CCDC190*	2.1	6.17 × 10^−9^
0	SLC1A3	0.39	7.95 × 10^−10^
0	LRRC32	0.4	2.15 × 10^−9^
0	PIK3IP1	0.45	6.59 × 10^−10^

Summary of RNA sequencing outputs identifying the greatest differentially expressed target genes using DeSeq2, ranked in the top 100 genes for fold change and adjusted *p* value comparing ES and ES-CSC RNA profiles. Bold = targets with increased expression in ES-CSCs chosen for quantification using RT-qPCR.* = targets confirmed as increased in ES-CSCs by RT-qPCR and further validated at the protein level by ICC and IHC using cell cultures and ES taken at diagnosis, respectively

RNA expression quantified by RT-qPCR (Additional file 8, Table S5) and from total RNA sequencing was found to be highly correlated; Neurexin-1 (NRXN1) R^2^ = 0.92, Extracellular Leucine Rich Repeat and Fibronectin Type III Domain Containing 2 (ELFN2) R^2^ = 0.87, Coiled-coil Domain Containing 190 (CCDC190) R^2^ = 0.78, Solute Carrier Family 38 Member 11 (SLC38A11) R^2^ = 0.90, Toll Like Receptor 4 (TLR4) R^2^ = 0.74, Piezo Type Mechanosensitive Ion Channel Component 2 (PIEZO2) R^2^ = 0.93. Furthermore, the median expression of 5/6 of these RNAs was increased in the ES-CSC populations compared to the parental ES cell cultures, validating the approach we have taken to identify genes increased in ES-CSCs. Expression of neurexin-1 was elevated 13 fold (*p* < 0.001, Fig. 2C), ELFN2 34 fold (*p* < 0.001, Fig. 2D), SLC38A11 9 fold (*p* < 0.0001, Fig. 2E), CCDC190 4 fold (*p* = 0.0002, Fig. 2F) and TLR4 1.9 fold (*p* = 0.0007, Fig. 2G) in the ES-CSCs compared to parental patient-derived primary ES cells. As the fold increase or decrease in expression of PIEZO2 and TLR4 messenger RNAs (mRNAs), respectively, was less than 2-fold, PIEZO2 and TLR4 were excluded from further downstream analyses.

**Fig. 3:**
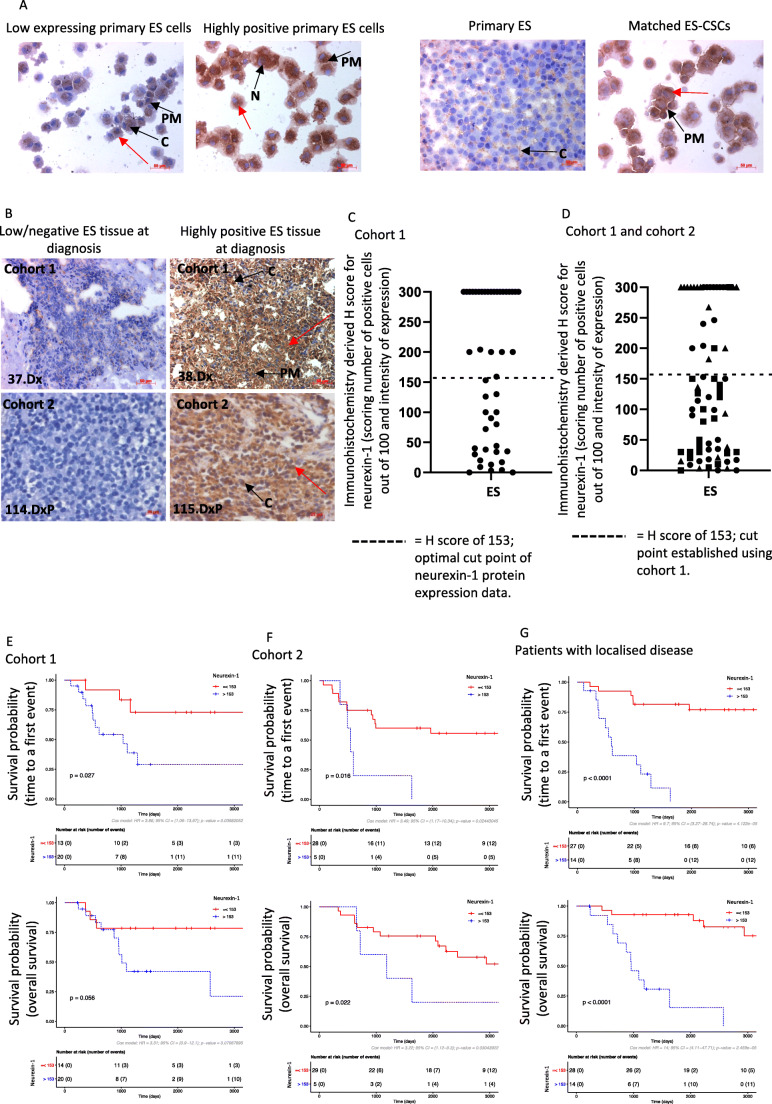
**Expression and prognostic potential of neurexin-1.** (**A**) Expression of neurexin-1 protein detected by ICC in patient-derived ES cultures and matched primary ES and ES-CSC. (**B**) IHC of neurexin-1 expression in ES taken at diagnosis (frozen tumour = cohort 1, FFPE = cohort 2). Representative images demonstrating low/negative tissues (tumour 37.diagnosis (Dx) and 114.Dx) and highly positive tissues (tumour 28.Dx and 115Dx); neurexin-1 protein detected by IHC using antibody ABN161-I (Millipore). Nuclei are labelled with haematoxylin. Red arrows = positive staining, black arrows = subcellular localisation of protein, C = cytoplasmic, N = nuclear, PM = plasma membrane. (**C**) Summary of neurexin-1 expression in ES taken at diagnosis (cohort 1) reported using H-score. Dashed line = H-score of 153, which is the cut-off point with highest concordance index in the Cox model. (**D**) Expression of neurexin-1 in ES tissue taken at diagnosis (cohort 1 and cohort 2). Dashed line = H-score of 153 which is the cut-off point established using cohort 1. Triangle = patients with localised disease at diagnosis who subsequently relapsed (*n* = 19), square = patients with localised disease at diagnosis who did not relapse (*n* = 22), circle = patients with metastasis at diagnosis (*n* = 27). Kaplan-Meier plots of the time to a first event and overall survival in days and hazard ratio based on neurexin-1 protein expression detected by IHC in (**E**) cohort 1, (**F**) cohort 2 and (**G**) patients with localised disease at diagnosis. Hazard ratios based on neurexin-1 protein expression dichotomising H-scores based on the cut-off point determined in the initial cohort (H-score = 153). Red line = neurexin-1 H-score ≤ 153, blue line = neurexin-1 H-score > 153

Expression of the remaining four candidate genes was confirmed at the protein level by ICC in patient-derived paired primary ES and ES-CSCs. Positive expression and subcellular localisation of neurexin-1 (100%, cytoplasmic and nuclear expression, H-score = 212 ± 39, range 80–300; Fig. 3A), ELFN2 (97%, plasma membrane, cytoplasmic and nuclear H-score = 219 ± 52, range 0–300), CCDC190 (85%, intracellular, H-score = 39 ± 20, range 0–100) and SLC38A11 (85%, plasma membrane and cytoplasmic, H-score = 87 ± 49, range 0–300) was confirmed in ES cultures (Table 3 and Additional file 8, Table S5). At the protein level neurexin-1 was most significantly increased in ES-CSCs compared to parental ES cells (*p* = 0.02; Fig. 3A, B).

**Table 3:** Differentially expressed genes in patient-derived primary ES and ES-CSCs that are associated with outcomes, including neurexin-1

Target gene identification method	Gene name	Protein name	Diagnosis ES: Cohort 1 protein expression				Surfaceome status	Diagnosis ES: GSE17618 RNA expression		Reported subcellular localisation
			Percentage of positive tumours	Mean H score ± s.d. (range)	Prognostic significance			Prognostic significance		
					EFS	OS		EFS	OS	
Differentially expressed genes comparing patient-derived paired ES and ES-CSCs (from top 50 genes for fold change and adjusted p value following DeSeq2)	CCDC190	Coiled-coil Domain Containing 190	67	41 ± 31 (0–300)	***KM p = 0.034, HR = 0.26 p = 0.046***	NS; KM p = 0.43, HR = 0.64 *p* = 0.43	non surface	NS	low exp.; p = 0.05	Intracellular vesicles
	ELFN2	Extracellular Leucine Rich Repeat and Fibronectin Type III Domain Containing 2	96	195 ± 53 (0–300)	NS; KM *p* = 0.21, HR = 0.48 p = 0.022	***KM p = 0.04, HR = 0.3 p = 0.052***	Gold	NS	NS	Plasma membrane, nucleus and intracellular vesicles
	NRXN1	Neurexin-1	96	***170 ± 61 (0–300)***	***KM p = 0.027, HR = 3.86 p = 0.039***	***KM p = 0.056, HR = 3.731 p = 0.071***	Gold	***KM p = 0.0093, HR = 2.71 p = 0.012***	***KM p = 0.027, HR = 2.5 p = 0.033***	Plasma membrane, nucleus and cytoplasm
	PIEZO2	Piezo Type Mechanosensitive Ion Channel Component 2	NA	NA	NA	NA	Silver	NS	NS	Plasma membrane, nucleus and intracellular vesicles
	SLC38A11	Solute Carrier Family 38 Member 11	41	16 ± 22 (0–200)	NS; KM *p* = 0.14, HR = 0.39 *p* = 0.16	NS; KM *p* = 0.092, HR = 0 *p* = 0.99	Silver	NS	NS	Plasma membrane
	TLR4	Toll like Receptor 4	NA	NA	NA	NA	Gold	NS	NS	Plasma membrane, golgi apparatus and cytoplasm
Neurexin-1 binding partners (identified from the literature)	APBA1	Amyloid Beta Precursor Protein Binding Family A Member 1	72	***98 ± 52 (0–300)***	***KM p = 0.0048, HR = 0.22 p = 0.009***	***KM p = 0.0019, HR = 0.2 p = 0.005***	non surface	KM p = 0.027, HR = 2.56 *p* = 0.032	KM = *p* = 0.017, HR = 2.83 p = 0.022	Golgi apparatus
	NLGN4X	Neuroligin 4 X-linked	100	***208 ± 49 (12–300)***	***KM p = 0.023, HR = 0.31 p = 0.030***	***KM p = 0.04, HR = 0.31 p = 0.05***	Gold	NS	NS	Plasma membrane
	NXPH3	Neurexophilin-3	41	30 ± 29 (0–300)	NS; (KM *p* = 0.22, HR = 2.53 *p* = 0.23)	NS; KM *p* = 0.15, HR = 2.33 p = 0.16	Silver	KM *p* = 0.049, HR = 1.97 *p* = 0.054	KM *p* = 0.0028, HR = 3.23 p = 0.004	Plasma membrane and secreted

The prognostic value of differentially expressed genes identified from RNA sequencing data, including neurexin-1 and neurexin-1 binding partners identified in the literature, were evaluated by IHC of tumour cohort 1 and interrogation of the publicly available GSE17618 RNA dataset. The cell surface expression of proteins graded using the ES surfaceome gene expression database [31] is shown; gold = known to be expressed at the cell surface, silver = predicted to be expressed at the cell surface

*NS*, not significant; *NA*, not analysed; *KM*, Kaplan-Meier; *HR*, Hazard ratio; bold italics = KM *p* value ≤ 0.05

Since neurexin-1α and neurexin-1β isoforms are transcribed from the same gene (NRXN1) [47], we went on to evaluate which isoforms were expressed in patient-derived primary ES cells by RT-qPCR. The dominant isoform was neurexin-1α (10/11 primary cell cultures; mean 2^-ΔΔCt^ = 0.00731, range 0.00002–0.01219, *p* = 0.02). However, since neurexin-1β was detected in 4/11 cultures (mean 2^-ΔΔCt^ = 0.06194, range 0.00224–0.72034) we decided to use a pan-neurexin-1 antibody which detects both neurexin-1α and neurexin-1β for Western blotting, IHC and ICC. Despite some evidence of redundancy in the neurexin family [48], neither neurexin-2 nor neurexin-3 were found to be differentially expressed in the ES-CSCs (adjusted *p* value > 0.001, results not shown).

Having confirmed increased expression of four candidate driver genes at the protein level in ES-CSCs, we examined their expression and potential association with outcome in a panel of ES tissues taken at diagnosis. All proteins were detected at the subcellular locations predicted from the surfaceome analysis of RNA sequencing data [31], reported in the literature and proteinatlas.org (Table 3). Most ES expressed neurexin-1 (96%) and ELFN2 (96%), whereas SLC38A11 and CCDC190 were detected in just 41% and 67% of the tumours examined, respectively (Table 3 and Additional file 8, Table S5).

### Association of candidate drivers of the ES-CSC phenotype with clinical outcome

We found that the expression of neurexin-1 in tumours was heterogeneous, localised to the plasma membrane, cytoplasm and occasionally the nucleus. Increased levels of expression were frequently observed in small groups of cells (Table 3, Fig. 3B and Additional file 9, Table S6). High neurexin-1 expression, defined as samples with a H-score > 153 (Fig. 3C), was associated with a reduced time to first event, predicting EFS (KM *p* = 0.027, HR = 3.86 HRp = 0.039) and related to a worse OS (KM *p* = 0.056, HR = 3.731 HR*p* = 0.071; Fig. 3E). In this initial dataset this was independent of patient age and presence of metastasis at diagnosis (HR = 2.0 HRp = 0.05), although there was some association with pelvic primary tumour site (HR = 0.9 HRp = 0.2), which is a known predictor of poor outcome in ES [25].

Expression of ELFN2 was also heterogeneous (Table 3 and Additional file 9, Table S6). Low expression of ELFN2 was significantly associated with a worse OS (KM *p* = 0.04, HR = 0.3 HR*p* = 0.052), but not with time to a first event (Table 3). CCDC190 and SLC38A11 (Table 3 and Additional file 9, Table S6) were only detected in single or small clusters of ES cells within the tumour, consistent with the hypothesis that these proteins are expressed by ES-CSCs and are, therefore, candidate therapeutic targets to eradicate ES-CSCs. However, SLC38A11 did not predict EFS or OS and, although low expression of CCDC190 was associated with EFS (KM *p* = 0.034, HR = 0.26 HRp = 0.046), it was not predictive of OS (Table 3). These observations require further investigation in a larger cohort.

### Validation of neurexin-1 as a prognostic factor

Neurexin-1 was the only prioritised target that, when expressed at high levels in tumours at diagnosis, was associated with an adverse outcome (EFS and OS). We therefore went on to evaluate the prognostic potential of neurexin-1 in a second tumour cohort. In this cohort FFPE ES were employed to test the suitability of neurexin-1 IHC for analysis in FFPE tumours. The pattern of neurexin-1 in FFPE tissues was similar to that in frozen tumours. High expression of neurexin-1 (89% ES positive, mean H-score = 87.9 ± 45; Fig. 3D and Additional file 9, Table S6) when dichotomising expression using the previously defined H-score of 153 remained predictive of EFS (KM *p* = 0.016, HR = 3.49 HRp = 0.02) and OS (KM *p* = 0.022, HR = 3.22 HRp = 0.03; Fig. 3F). In the publicly available RNA dataset of ES diagnosis tissues (GSE17618), neurexin-1 mRNA was also predictive of both EFS (KM *p* = 0.0093, HR = 2.71 HRp = 0.012) and OS (KM *p* = 0.027, HR = 2.5 HR*p* = 0.033).

### High neurexin-1 expression predicts relapse in patients with localised disease

Combining patients diagnosed with localised disease from tissue sample cohorts 1 and 2 revealed that neurexin-1 was predictive of EFS (*n* = 41, 18/41 relapsed, KM *p* < 0.0001, HR = 9.7 HRp < 0.0001) and OS (*n* = 42, KM *p* < 0.0001, HR = 14 HRp < 0.0001, Fig. 3G). For those patients with localised disease who have not had an event to date, 91% (20/23) had low neurexin-1 expression and a mean EFS of > 7 years. These observations suggest that neurexin-1 expression in tumours at diagnosis could be used for the early identification of patients with localised disease that relapse and may benefit from more intensive treatment or rapid transfer to early phase clinical trials of novel agents.

### Functional evaluation of neurexin-1 using shRNA and siRNA

To investigate the functional relevance of neurexin-1 we first employed shRNA targeting of the NRXN1 gene to reduce the expression of neurexin-1 in two patient-derived primary ES cultures, parental.17 and parental.23. Cells were also infected with a scramble shRNA, which was included in all experiments as a control (shControl).

**Fig. 4:**
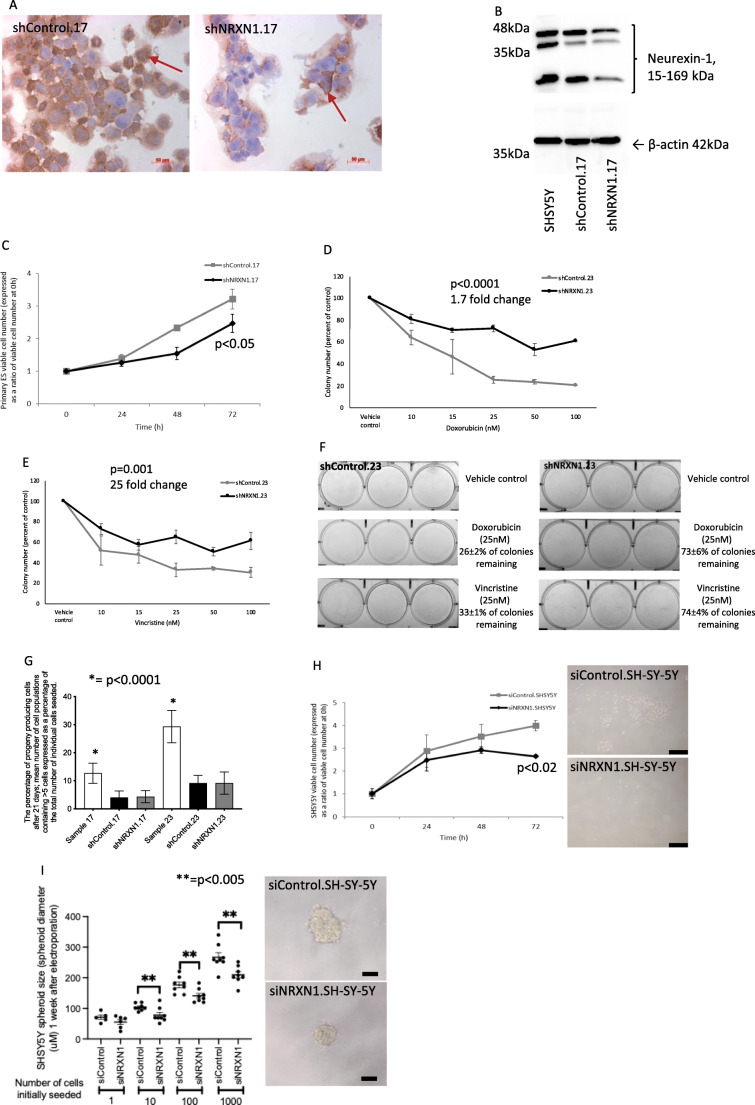
**Reduced neurexin-1 expression decreases cell viability, induces resistance to doxorubicin and vincristine and decreases the formation of 3D spheroids.** (**A**) Expression of neurexin-1 detected by ICC in shControl and shNRXN1 primary ES cells. Red arrows = plasma membrane neurexin-1 expression. (**B**) Western blot showing high expression of neurexin-1 in SH-SY-5Y positive control cells and partial knock-down of neurexin-1 protein in shNRXN1 treated cells compared to shControl cells. Equal protein loading was confirmed by probing the Western blot for expression of β-actin. (**C**) The number of viable cells after shNRXN1 knock-down compared to shControl cells over 72 h was significantly decreased. Viable cell numbers were quantified using a trypan blue exclusion assay; viable cell numbers are presented as ratio of the viable cell number at 0 h. Results are given as mean ± SEM, for 3 independent experiments. Knockdown of neurexin-1 by shNRXN1 decreased the sensitivity of ES clones to (**D**) doxorubicin (10–100 nM) or (**E**) vincristine (10–100 nM). In (**D**) and (**E**) colonies were incubated with cytotoxic chemotherapy (0–100 nM) for 72 h, washed and maintained in appropriate media for another 7 days before fixing, staining with crystal violet and counting. (**F**) Representative images of shControl and shNRXN1 derived colonies after incubation with vehicle control, doxorubicin (25 nM) or vincristine (25 nM). (**G**) Effect of shNRXN1 and shControl on single cell self-renewal after 21 days. Clones contained > 5 cells and are expressed as percentage of the total number of individual cells seeded; results are shown as mean ± SEM. Infection with shNRXN1 or shControl significantly decreased the colony forming capacity of both patient-derived cultures (patient.17 and patient.23). (**H**) Decreased number of viable SH-SY-5Y cells after infection with siNRXN1 compared to control siRNA treated cells over 72 h. Viable cell numbers are presented as ratio of the viable cell number at 0 h. Results are given as mean ± SEM. SH-SY-5Y siControl and siNRXN1 cells grew as monolayers and did not form colonies. Representative images showing decreases in siNRXN1 SH-SY-5Y cell numbers following culture in adherent conditions for 10 days compared to siControl cells (scale bar = 500 μm). (**I**) The sizes of SH-SY-5Y spheroids formed from siNRXN1 cells were significantly decreased compared to those from siControl cells under low adherence conditions. Representative images of siNRXN1 and siControl derived SH-SY-5Y spheroids are shown (scale bar = 100 μm)

Decreased expression of neurexin-1 was confirmed by ICC of cells (Fig. 4A) and Western blotting of proteins extracted from shNRXN1 and shControl infected cells (Fig. 4B). The partial shRNA knock-down of neurexin-1 was associated with a decrease in shNRXN1.17 and shNRXN1.23 viable cell numbers (fold change in shNRXN1 cell number over 72 h = 2.46 ± 0.2 and 1.42 ± 0.08, respectively) compared to control infected cells (fold change in shControl cell number over 72 h = 3.21 ± 0.3 and 1.7 ± 0.08, respectively; *p* < 0.05, Fig. 4C). Cells with reduced levels of neurexin-1 protein (shNRXN1 cells) compared to control cells (shControl) were also more resistant to the cytotoxicity of doxorubicin (EC50 = 19 nM and 11 nM, respectively, 1.7 fold change in resistance, *p* < 0.0001, Fig. 4D and F) and vincristine (EC50 = 8 nM and 0.3 nM, > 25 fold change in resistance, *p* = 0.001, Fig. 4E and F). This increase in resistance may correlate with reduced cell cycle progression and viable cell numbers in shNRXN1 cells compared to shControl cells, although it did not correlate with cell proliferation, which was not statistically different in shNRXN1 (shNRXN1.17 20 ± 0.3%, shNRXN1.23 26 ± 0.8%) and shControl (shControl.17 17 ± 1%, shControl.23 26 ± 0.2%) cells. Together these observations suggest that neurexin-1 plays a role in ES cell survival and/or cell death pathways, which may trigger responses to standard-of-care chemotherapeutics independent of proliferation. This notion requires further investigation.

To evaluate the potential role of neurexin-1 in single cell self-renewal, we wanted to use long-term stable shNRXN1 knock-down of patient-derived primary ES cells. Infection of cultures parental.17 and parental.23 with shNRXN1 or shControl significantly reduced the self-renewing capacity of both cultures compared to noninfected cells at 21 days (Fig. 4G; *p* < 0.0001), suggesting that off-target activities of shRNA, viral infection and/or incubation with puromycin reduces the single cell self-renewing capacity of primary ES cells. Single cell self-renewal of ES cells in this substrate-adherent 21 day assay, after partial knock-down of neurexin-1, was not significantly different to that in shControl infected cells (Fig. 4G), likely reflecting decreased viability of patient-derived primary ES cells after infection with shRNA.

To investigate whether neurexin-1 has a functional role in self-renewal, we used siRNA to decrease expression in SH-SY-5Y cells, which expresses high levels of neurexin-1. The viability of SH-SY-5Y cells was not affected by the optimised electroporation conditions, and partial knock-down (1.6 to 1.4 fold; *p* < 0.0001) of neurexin-1 was confirmed by RT-qPCR up to 72 h in siNRXN1 compared to siControl electroporated cells. Consistent with the decreased viable cell number in shNRXN1 patient-derived primary ES cells compared to shControl, siRNA knockdown of neurexin-1 in SH-SY-5Y cells significantly decreased the viable cell number (fold change in siNRXN1 cell number over 72 h = 2.64 ± 0.05) compared to siRNA control electroporated cells (fold change in siControl cell number over 72 h = 3.99 ± 0.22; *p* < 0.02, Fig. 4H). As SH-SY-5Y cells do not form colonies in our adherent self-renewing assay, we investigated the potential role of neurexin-1 in self-renewal using a 3D spheroid assay. Importantly, we found that spheroids formed in 100% of wells following seeding of 10, 100 or 1000 siControl or siNRXN1 SH-SY-5Y cells. siNRXN1 knockdown, however, decreased the spheroid sizes compared to those of siControl cells (*p* < 0.005, Fig. 4H), consistent with the hypothesis that neurexin-1 plays a role in the self-renewal and growth of 3D spheroids.

These studies show that neurexin-1 can affect drug resistance and 3D spheroid formation, consistent with a putative role in ES-CSCs and highlighting the importance of investigating the potential biological relevance of the neurexin-1 pathway in ES.

### Neurexin-1 binding partners predict ES patient outcome

**Fig. 5:**
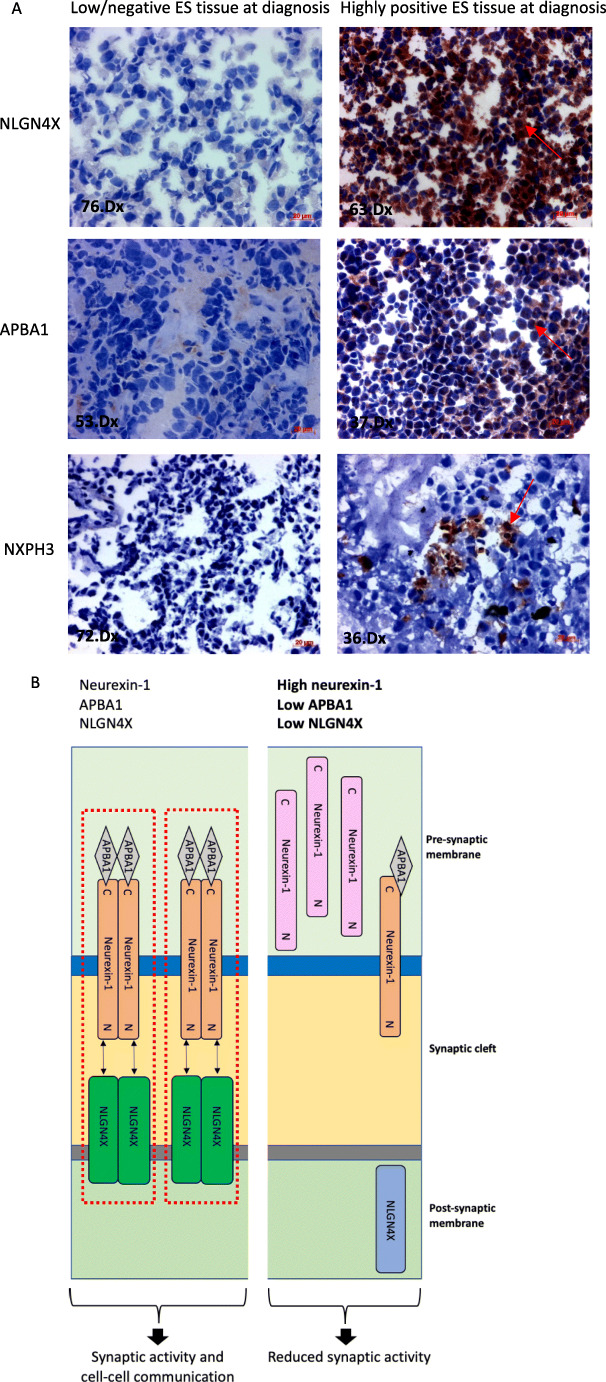
**Expression of neurexin-1 binding partners and summary of their putative roles in ES.** (**A**) IHC of NLGN4X, APBA1 and NXPH3 in ES taken at diagnosis; representative images of low and high expressing tumours are shown. Red arrows = positive expression. (**B**) Summary of proteins that bind and transport neurexin-1 to the pre-synaptic membrane (ABPA1) and bind the pre-synaptic complex to induce post-synaptic membrane activity (NLGN4X). Bold = levels of neurexin-1 and associated binding partners identified in patients with poor outcome; high expression of neurexin-1 and low levels of APBA1 and NLGN4X result in reduced synaptic activity. NLGN4X = Neuroligin 4 X-linked. APBA1 = Ameloid Beta Precursor Protein Binding Family A Member 1. Neurexin-1 orange = Neurexin-1 bound to APBA1. Neurexin-1 pink = Unbound neurexin-1. NLGN4X green = NLGN4X bound to neurexin-1-APBA1 complex. NLGN4X blue = Unbound NLGN4X. C = C-terminal/cytoplasmic domain and N = N-terminal of the neurexin-1 protein. Black bi-directional arrow = binding of APBA1-Neurexin-1 complex and NLGN4X across synaptic cleft. Red dotted line = formation of APBA1-Neurexin-1-NLGN4X hetero-tetramer

Since expression of neurexin-1 was found to be associated with outcomes in ES, we went on to investigate the expression of neurexin-1 binding partners that regulate its subcellular localisation and synaptic activity in tumour cohort 1, i.e., Amyloid Beta Precursor Protein Binding Family A Member 1 (APBA1), Neuroligin 4 X-linked (NLGN4X) and Neurexophilin 3 (NXPH3). All three proteins were differentially expressed in the cell membrane and cytoplasm of diagnosis tumours (Table 3, Fig. 5A and Additional file 9, Table S6). Low expression of APBA1 or NLGN4X was associated with poor EFS and OS rates, dichotomising the data using the optimal cut-off points and H-scores of 6 (EFS, KM *p* = 0.0048, HR = 0.22 HRp = 0.009 and OS, KM *p* = 0.0019, HR = 0.2 HR*p* = 0.005) and 184 (EFS, KM *p* = 0.023, HR = 0.31 HR*p* = 0.030 and OS, KM *p* = 0.04, HR = 0.31 HR*p* = 0.05), respectively (Table 3). NXPH3 protein was detected in single or small groups of cells within ES tumours, but was not associated with EFS or OS in this small cohort (Table 3 and Additional file 9, Table S6).

High expression of neurexin-1 and low expression of NLGN4X identified the same patients in 26/44 cases (59%), although the prognostic significance of NLGN4X was independent of neurexin-1 (EFS *p* = 0.02 and OS *p* = 0.05). This likely reflects the multiple binding partners of neurexin-1 compared to the more limited interactions of NLGN4X [49]. Like neurexin-1, the prognostic significance of NLGN4X appeared to be independent of patient age and metastasis (EFS, HR = 2.4, HRp = 0.01 and OS, HR = 2.5 HRp = 0.01).

## Discussion

Using transcriptomic approaches, we revealed the surfaceome of patient-derived ES primary cell cultures and ES-CSCs and, by doing so, identified candidate biomarkers of risk and therapeutic targets. We found that the synaptic adhesion protein neurexin-1, and regulators of this pathway, were associated with ES outcome. Neurexin-1 was found to be involved in self-renewal and drug resistance, characteristics of CSCs. Consistent with our hypothesis that ES-CSCs drive disease progression and relapse, high expression of neurexin-1 defined a previously unidentified group of patients with localised disease at diagnosis who develop metastasis and relapse. Supporting a role of the neurexin-1 pathway in ES progression and relapse, reducing the expression of neurexin-1 increased drug resistance independent of cell proliferation and decreased 3D spheroid formation and growth. In addition, we found that low expression of the neurexin-1 binding partners APBA1 and NLGN4X were also associated with poor clinical outcomes, consistent with the reported roles of this pathway in cell migration, cell-cell adhesion and cell survival [50].

Parental patient-derived primary ES cultures and daughter ES-CSCs share the transcriptional fingerprint of ESCs [42, 43] and MSCs [13, 44], consistent with the mesenchymal origin of ES [51]. Furthermore, these cells retain the EWSR1-ETS fusion transcript and cell surface expression of CD99 with the tumours from which they were derived [11, 52]. Of the most highly expressed RNAs in patient-derived cells with a predicted cell surface localisation and low expression in normal tissues, FBN1 was the highest ranked one. FBN1 is an EWSR1-friend leukaemia integration 1 transcription factor (FLI1) target gene [53], encoding a large protein called fibrillin-1. Fibrillin-1 is secreted by cells into the extracellular matrix where it binds with other proteins to form microfibrils, which provide support to many structures including bones and tissues in which ES can arise. High expression of fibrillin-1 has previously been reported in ES cell lines [54] and to promote tumorigenesis and metastasis in some cancers, including ovarian [55] and colorectal cancer [56].

The most highly expressed RNAs in ES and ES-CSCs identified in this study play putative roles in the regulation of cell-extracellular matrix interactions, consistent with the function of the bone microenvironment in tumour initiation and progression [57]. Their low expression in normal tissues and association with an adverse outcome or phenotype, validates the strategy we have taken to identify candidate therapeutic targets. Forty percent (4/10) of the highly expressed cell surface proteins were members of the collagen gene family, previously reported to be upregulated in ES [58]. Integrin subunit beta 1 (ITGB1) and galectin 1 (LGALS1), which form complexes with collagens and regulate the structure and interactions of cells with the extracellular matrix [59], were also highly expressed in patient-derived primary ES cells. Both ITGB1 and LGALS1 mediate cell proliferation, migration and tumour progression [60, 61]. High expression of ITBG1 protein has also been linked to a worse outcome in a range of adult cancers [62]. Serpine2, which we found to be highly expressed in ES and ES-CSCs, is also highly expressed in colorectal and breast cancers, where it is reported to promote lymph node metastasis [63]. Furthermore, the activity of Insulin like Growth Factor Binding Protein 4 (IGFBP4), which was also highly expressed in ES and ES-CSCs, is regulated by proteins of the tumour microenvironment which may include fibrillin-1 [64]. In ES, EWSR1-FLI1 induces pappalysin-1 expression cleaving IGFBP4, releasing insulin growth factor (IGF) signalling from inhibition to increase growth and tumorigenicity [65]. This is consistent with the established role of insulin growth factor signalling in the pathogenesis and progression of ES [66, 67].

Although previous studies have identified candidate prognostic or therapeutic targets using Next Generation Sequencing (NGS) technologies, this is the first report describing RNA expression profiling of patient-derived paired ES primary cell cultures and ES-CSCs. Consistent with our observations, RNA sequencing of 13 ES has previously resulted in the identification of extracellular matrix genes to predict adverse outcomes [67], underscoring the importance of extracellular matrix and tumour microenvironment interactions in ES. Interrogation of our dataset confirmed the expression of genes including those encoding urotensin 2, insulin-like growth factor 2 and periostin [67] in ES and ES-CSCs (Additional file 7, Table S4), although these were expressed at lower levels than the top cell surface genes fibrillin 1, collagen type VI alpha 3 chain and collagen type XII alpha 1 chain identified using our pipeline. Periostin was, however, expressed at high levels in patient-derived primary ES and ES-CSCs, where it may regulate matrix proteins and ES cell-matrix interactions [68]. High expression of the fibroblast growth factor receptor 1 (FGFR1) gene has also been associated with relapse in ES [68]. Although FGFR1 mRNA was detected at reasonable levels in ES and ES-CSCs (Additional file 7, Table S4), it is also expressed in a range of normal tissues, which may limit its therapeutic potential. Supporting the role of prominin-1 (CD133) as a marker of ES-CSCs and prognostic indicator [11], RNA expression of prominin-1 mRNA in the GSE 17613 dataset was found to be associated with poor outcome. However, it was rarely detected in our patient-derived ES cells. On the other hand, although low levels of ALDH were detected by RNA sequencing in the patient-derived ES cells, its high expression did not predict outcome in the GSE17618 dataset. In contrast, read counts and prognostic significance of the ABC transporter proteins P-glycoprotein (Pgp) and multi-drug resistance protein-1 (MRP1) (Additional file 6; Table S3), were consistent with previous observations reporting the prognostic significance of high levels of MRP1 [37, 46], but not Pgp [46] in ES. These observations emphasise the importance of comparing the prognostic and functional significance of candidate biomarkers of risk with established clinical risk factors at diagnosis, to develop a high performance prognostic model combining biology and clinical risk factors to predict patient outcomes to meet personalised medical needs.

Of the 4 candidates most significantly differentially expressed and validated in the paired ES and ES-CSC samples, neurexin-1 was the target gene with highest cell surface protein expression that was associated with survival. Identification of this novel prognostic biomarker validates the transcriptomics approach we have taken. However, a limitation of this strategy is loss of genes that are differentially expressed at low levels but have substantial biological activity. The prognostic potential of neurexin-1 expression in FFPE and frozen tumours demonstrates its suitability for transfer to the clinic, where it may be used at diagnosis to identify patients with localised disease that will develop metastasis and relapse [6] for more intensive or novel treatment. The independent prognostic value of neurexin-1 is currently being compared to other adverse prognostic factors in an international collaboration, including gain of chromosome 1q and loss of 16q [69, 70], cell-cycle and proliferation regulation [70–73], stromal antigen 2 (STAG2) and p53 [22–24] and expression of insulin like growth factor binding protein 3 (IGF2BP3) [74].

High expression of neurexin-1 has been linked with carcinogenesis, invasion and proliferation in breast cancer [75], drug response in gastric cancer [76] and to be predictive of a worse outcome in oral squamous cell carcinoma [77]. Supporting these observations, we found that neurexin-1 expression predicts outcome in patients and affects 3D spheroid formation and resistance to cytotoxic chemotherapeutics. We are currently investigating the biological relevance of the neurexin-1 pathway in ES-CSCs using single cell transcriptomics and high-content imaging, and multicellular preclinical models to investigate its role in cell survival, cell death and the development of bone metastasis [78]. Deletion of the neurexin-1 gene alters synapse function and neuronal connectivity in astrocytes, leading to inhibition of differentiation [79], supporting the established role of neurexin-1 in neurodevelopmental diseases such as schizophrenia, Tourette syndrome, epilepsy and autistic spectrum disorder [49]. In normal cells, neurexin-1 expression is restricted primarily to the pre-synaptic nerve terminal of the brain where it is an essential regulator of synapse properties [80]. A diverse mix of downstream neurexin-1 proteins is translated as a result of alternative splicing and transcription through isoform-specific promoters. These alternative transcriptional start sites produce neurexin-1α (1477 amino acids) and the shorter neurexin-1β (472 amino acids) proteins which are reported to have distinct functional activities and expression profiles [48, 49]. Both proteins can be presented on the surface of the pre-synaptic membrane following binding to APBA1 (Fig. 5B), although neurexin-1α preferentially binds neurexophilins such as NXPH3 and neurexin-1β to neuroligins such as NLGN4X in the post-synaptic membrane [49]. Consistent with the role of the neurexin-1 pathway in ES, we found that low expression of the neurexin-1 binding partners APBA1 and NLGN4X was associated with a poor outcome. Tripartite expression of neurexin-1, APBA1 and NLGN4X may be required for cell-cell communication in ES, neurexin-1 being presented as a hetero-tetramer on the pre-synaptic membrane by APBA1 and binding with NLGN4X on the post-synaptic membrane (Fig. 5B). Since neurexin-1 dependent pathways regulate pre- and post-synaptic organisation, cell migration, motility and cell-cell adhesion [50, 80, 81], we are currently investigating their role in multicellular models and tumours. We are also evaluating the independent prognostic value of neurexin-1 protein and regulators of its pathway. Whether neurexin-1 can be exploited therapeutically, by targeting the neurexin-1 pathway or by targeted delivery of small molecules to improve outcomes for some patients remains to be seen.

## Supplementary Information

ESM 1 (DOCX 19 kb)

ESM 2 (DOCX 12 kb)

ESM 3 (DOCX 14 kb)

ESM 4 (XLSX 13 kb)

ESM 5 (DOCX 13 kb)

ESM 6 (XLSX 13 kb)

ESM 7 (PDF 419 kb)

ESM 8 (XLSX 12 kb)

ESM 9 (XLSX 14 kb)

ESM 10 (XLSX 16 kb)

## Data Availability

All data generated or analysed during this study are included in this article and its accompanying additional information files. The FASTQ files are available in the Research Data Leeds Repository (University of Leeds), Burchill, Susan and Roundhill, Elizabeth (2020): Total RNA sequencing of patient-derived Ewing sarcoma and Ewing sarcoma CSCs University of Leeds. [Dataset]. 10.5518/887

## List of abbreviations

ABC: ATP-binding cassette
ANOVA: Analysis of variance
APBA1: Ameloid Beta Precursor Protein Binding Family A Member 1
bp: Base pairs
C: Cytoplasmic
CCDC190: Coiled-coil Domain Containing 190
CD99: CD99 antigen
CD117: c-Kit
CD133: Prominin-1
CD271: Low affinity Nerve Growth Factor Receptor
CFSE: Carboxyfluorescein Succinimidyl Ester
CLIC4: Chloride intracellular channel 4
COL6A1: Collagen type VI Alpha 1 chain
COL6A2: Collagen type VI Alpha 2 chain
COL6A3: Collagen type VI Alpha 3 chain
COL12A1: Collagen type XII Alpha 1 chain
CSC: Cancer stem-like cell
Dx: Diagnosis tissue
DxP: FFPE Diagnosis tissue
EDTA: Ethylenediaminetetraacetic acid
EFS: Event Free Survival
ELFN2: Extracellular Leucine Rich Repeat and Fibronectin Type III Domain Containing 2
ETS: Erythroblast Transformation Specific
ES: Ewing sarcoma
ESC: Embryonic Stem Cell
EWSR1: Ewing Sarcoma Breakpoint Region 1
FBN1: Fibrillin 1
FGFR1: Fibroblast Growth Factor Receptor 1
FISH: Fluorescent In-Situ Hybridization
FFPE: Formalin Fixed, Paraffin Embedded
FLI1: Friend Leukaemia Integration 1 transcription factor
GO: Gene ontology
HR: Hazard Ratio
HRp: Hazard ratio *p* value
ICC: Immunocytochemistry
ICS: Interaction Confidence Score
IGF: Insulin Growth Factor
IGFBP3: Insulin like Growth Factor Binding Protein 3
IGFBP4: Insulin like Growth Factor Binding Protein 4
IHC: Immunohistochemistry
ITGB1: Integrin subunit Beta 1
KM: Kaplan Meier
LGALS1: Galectin 1
LRP1: LDL Receptor related Protein 1
LRRC32: Leucine Rich Repeat Containing 32
MDR: Multi-drug resistant
MI: Migration Index
mRNA: Messenger RNA
MSC: Mesenchymal Stem Cell
N: Nuclear
NA: Sample not analysed
NGS: Next Generation Sequencing
NI: No interaction reported in STRING database
NLGN4X: Neuroligin 4 X-linked
NRXN1: Neurexin-1
NS: Not significant
NXPH3: Neurexophilin 3
OCT: Optimum Cutting Temperature compound
OCT4: Octamer-binding transcription factor 4
OS: Overall Survival
PBS: Phosphate buffered saline
PIEZO2: Piezo Type Mechanosensitive Ion Channel Component 2
PIK3IP1: Phosphoinositide-3-Kinase Interacting Protein 1
PM: Plasma membrane
PPIA: Peptidylprolyl Isomerase A
REMARK: Reporting Recommendation for Tumor Marker Prognostic Studies
RNA: Ribonucleic acid
RT-PCR: Reverse-Transcriptase Polymerase Chain Reaction
RTqPCR: Reverse-Transcriptase quantitative Polymerase Chain Reaction
s.d.: Standard deviation
SEM: Standard Error of the Mean
Serpine 2: Serpine family E member 2
shRNA: Short hairpin RNA
SLC1A3: Solute Carrier Family 1 Member 3
SLC38A11: Solute Carrier Family 38 Member 11
SR: Self-Renewal
STAG2: Stromal Antigen 2
STAR: Spliced Transcripts Alignment to a Reference
STRING: Search Tool for Retrieval Interacting Genes/proteins
TLR4: Toll Like Receptor 4
