# Supplementary material for: RNA sequencing and functional studies of patient-derived cells reveal that neurexin-1 and regulators of this pathway are associated with poor outcomes in Ewing sarcoma
Source: Cell Oncol (Dordr). 2021 Aug 17;44(5):1065–85. doi: 10.1007/s13402-021-00619-8 (PMC8516792; doi:10.1007/s13402-021-00619-8)
Supplement: Supplementary file 1 — (DOCX 19 kb) [file 13402_2021_619_MOESM1_ESM.docx]

**Additional files**

**Additional file 1. Data S1.**

File format: Additional file 1_Data S1.docx

Title: Cell lines

Description of data: Culture and cell line information.

**Additional file 2. Data S2.**

File format: Additional file 2_Data S2.docx

Title: Methods to characterise ES cells: FISH, RT-PCR, ICC and Western blot.

Description of data: Method details for fluorescence in situ hybridisation (FISH), reverse transcriptase polymerase chain reaction (RT-PCR), preparation of cytospins for immunocytochemistry (ICC), immunocytochemistry for CD99, Western blotting.

**Additional file 3. Table S1.**

File format: Additional file 3_Table S1.xlsx

Title: RTqPCR and ICC methods used to quantify and validate the top differentially expressed genes.

Description of data: *Reverse transcriptase quantitative polymerase chain reaction (RTqPCR).* For the analysis of mRNA target expression by RTqPCR total RNA (10ng) was reverse transcribed and cDNA added to sequence specific reverse and forward primers and probe for the endogenous control gene peptidylprolyl isomerase A (PPIA), single target assay on demand for target genes (Thermo Fisher Scientific; Additional file 3, Table S1) or NRXN1-α and NRXN1-β and 1 x TaqMan® Universal PCR Master Mix (Invitrogen). Expression of mRNA was calculated using the comparative Ct method, relative to the endogenous control gene PPIA and the reference control cell line [21]. To ensure the specificity of primer and probe combinations targeting NRXN1-α and NRXN1- β and associated alternative splicing events, the predicted size of the resulting full-length amplicons (167bp and 144bp respectively) from the RTqPCR reaction were confirmed by agarose gel electrophoresis. Although NRXN1-α appears to be the dominant NRXN1 isoform in the ES cell cultures examined, NRXN1-β expression was also detected. Therefore, an NRXN1 antibody that binds both isoforms was used in subsequent studies.

*Immunocytochemistry (ICC)*. Protein expression of target genes on cytospins of ES and ES-CSCs (Additional file 3, Table S1) was detected using the species appropriate EnVision+ System-HRP (DAB) kit (Dako) and target specific or corresponding isotype control antibodies (4μg/ml, Negative Control Mouse IgG1, X0931 (Dako) and 20μg/μl, Normal Rabbit Serum Control Ig mix, 086199 (Life Technologies)). Optimum antibody concentrations were determined empirically using positive control cell lines. Cells were visualised using the Liquid DAB Substrate Chromogen System for peroxidase (Dako), counter-stained with 0.1% Mayer’s haematoxylin at room temperature for 15s. Cells were visualised by light microscopy (Zeiss Axioplan microscope). Protein expression of each target was scored manually by two independent reviewers. All samples were scored using the H-score [36] taking into account the number of positive cells and intensity of expression (low (+), medium (++) and high (+++)).

**Additional file 4. Data S3.**

File format: Additional file 4_Data S3.docx

Title: Method to evaluate the prognostic potential of differentially expressed proteins.

Description of data: Methodology for the preparation of tumour sections for IHC and the IHC procedures.

**Additional file 5. Table S2.**

File format: Additional file 5_Table S2.xlsx

Title: Summary of ES cell characteristics, tumours and patients.

Description of data: Typical or atypical cytology, *EWSR1* gene fusion status, CD99 expression, those analysed by total RNA sequencing, progeny producing ability, response to therapy, migration and matched clinical information, such as time to a first event, event status, follow up time, current status and presence of metastasis at diagnosis for primary ES cultures examined in this study. NA=not analysed. The median age at diagnosis was 12 years; range 5-20 years. The median follow up time and time to a first event was 912 and 501 days respectively, with 57% of patients having an adverse event which could be relapse or death due to disease. Patients without an event are censored at the date the patient was last seen.

**Additional file 6. Table S3.**

File format: Additional file 6_Table S3.xlsx

Title: MSC, ESC and CSC associated genes identified by RNA sequencing of primary patient-derived ES cultures.

Description of data: Genes associated with mesenchymal stem cells (MSC), embryonic stem cells (ESC) and the cancer stem-like cell phenotype (CSC) with the corresponding mean (±s.d.) read counts of these genes across primary patient-derived ES cultures generated by total RNA sequencing. The prognostic significance (event free survival (EFS) and overall survival (OS)) of high target gene RNA expression from the online database GSE17618 are also listed. NS=not significant, KM = Kaplan Meier, HR = Hazard ratio, *p* = probability.

*Detection and quantification of CD99 RNA.* Since CD99 is expressed on the pseudo-autosomal region of both chromosomes X and Y, CD99 reads would ordinarily be discarded as a result of multiple alignment possibilities. Therefore, a gene specific reference was generated which included ± 1000 bases either side of CD99 (ENSG00000002586), accounting for any possible flanking of read pairs. Post-trimmed reads were aligned using STAR to this CD99 gene specific reference and the number of CD99 raw reads per sample generated.

**Additional file 7. Table S4.**

File format: Additional file 7_Table S4.xlsx

Title: Candidate prognostic or therapeutic target genes previously identified in ES using NGS technologies.

Description of data: RNA sequencing data from patient-derived ES and ES-CSCs reported in this paper, was interrogated for the expression of genes identified using NGS technologies as candidate prognostic or therapeutic targets. The mean RNA read count (±s.d.) and rank of genes determined by total RNA sequencing of ES and ES-CSCs in the current study are listed. To determine the cell surface localisation of genes, a surfaceome database (www.imm.ox.ac.uk/research/units-and-centres/mrc-molecular-haematology-unit/research-groups/rabbitts-group/more-from-the-rabbitts-group/surfaceome-database) was interrogated in which RNA species are classified as gold, silver or bronze where a classification of gold represents a protein with known cell surface expression; gold (known cell surface such as CD markers), silver (multiple independent feature predictions or annotations), and bronze (single feature prediction or annotation) [34].

**Additional file 8. Table S5.**

File format: Additional file 8_Table S5.xlsx

Title: RNA and protein expression of candidate driver genes in patient-derived ES and ES-CSCs.

Description of data: RNA expression of genes identified for further validation by RTqPCR (reported as 2-ΔΔCt; analysing 10ng of RNA, normalising Ct values to the endogenous control gene PPIA and the appropriate control cell line). Protein expression of target driver genes using immunocytochemistry (ICC; reported using the H-score) in primary ES and ES-CSCs. H-score=number of positive cells multiplied by the intensity of expression (low (+=1), medium (++=2) and high (+++=3)). NA= sample not examined.

**Additional file 9. Table S6.**

File format: Additional file 9_Table S6.xlsx

Title: Summary of patient cohorts and profile of candidate driver genes.

Description of data: Patient age, presence of metastasis at diagnosis, time to a first event, status, follow up time and protein expression of target genes detected using IHC (reported using the H-score), in an initial (.Dx suffix) and secondary cohort (.DxFFPE(P) suffix) of diagnosis ES are displayed. H-score=number of positive cells multiplied by the intensity of expression which could be low (+=1), medium (++=2) or high (+++=3) [36]. Each sample number denotes an independent tumour sample and patient. The median age of cohort 1 ES patients at diagnosis was 13 years; range 2-38 years. The median follow up time and time to a first event was 873 and 534 days respectively; 36% of patients had an adverse event. The median age of cohort 2 ES patients at diagnosis was 19 years; range 5-64 years. The median follow up time and time to a first event was 1906 and 1497 days respectively; 48% of patients had an adverse event. ES diagnosis was confirmed by pathology review. NA=sample not analysed or missing clinical information.
