# Supplementary material for: RNA sequencing and functional studies of patient-derived cells reveal that neurexin-1 and regulators of this pathway are associated with poor outcomes in Ewing sarcoma
Source: Cell Oncol (Dordr). 2021 Aug 17;44(5):1065–85. doi: 10.1007/s13402-021-00619-8 (PMC8516792; doi:10.1007/s13402-021-00619-8)
Supplement: Supplementary file 2 — (DOCX 12 kb) [file 13402_2021_619_MOESM2_ESM.docx]

**Additional file 1. Cell lines.**

The substrate adherent ES cells (RD-ES, SK-N-MC, TC-32, TTC 466), MCF-7 (breast adenocarcinoma) [Roundhill, E.A. and S.A. Burchill, British journal of Cancer, 2012. **106**(6): p. 1224-33] and the U-2 OS (osteosarcoma) cells [Roundhill, E.A., S. Jabri and S.A. Burchill, Cancer Lett, 2019. **453**: p. 142-157.] were cultured as previously described. All ES cell lines contain EWSR1 gene re-arrangements and express CD99 in the cell membrane, characteristic of ES. The HL60 (promyelocytic leukocytes; CRUK/ICRF cell repository), BE(2)C (neuroblastoma; gift from Professor M Haber, CCI, Australia) and SHSY5Y (neuroblastoma; gift from Professor C Redfern, University of Newcastle, UK) were cultured in DMEM (Sigma-Aldrich) containing 10% foetal calf serum (FCS) and 2mM glutamine. The HEPG2 (hepatocellular carcinoma) cells were cultured in Modified Eagle Medium containing 10% FCS and 2mM glutamine (both a gift from Professor M Knowles). All cell lines are yeast, bacterial and mycoplasma-free; cultures are tested for mycoplasma every four months using the EZ-PCR mycoplasma test kit (Geneflow, UK).
