# Supplementary material for: RNA sequencing and functional studies of patient-derived cells reveal that neurexin-1 and regulators of this pathway are associated with poor outcomes in Ewing sarcoma
Source: Cell Oncol (Dordr). 2021 Aug 17;44(5):1065–85. doi: 10.1007/s13402-021-00619-8 (PMC8516792; doi:10.1007/s13402-021-00619-8)
Supplement: Supplementary file 3 — (DOCX 14 kb) [file 13402_2021_619_MOESM3_ESM.docx]

**Additional file 2. Methods to characterise ES cells: FISH, RT-PCR, ICC and Western blot.**

*Fluorescence in situ hybridisation (FISH).* Cells were fixed in methanol:acetic acid (3:1; 20min at room temperature) and incubated at 60°C for 1h. Cellular DNA was denatured (pH 7; 70% formamide (Fisher Scientific) in 2xSSC Buffer (3M sodium chloride, 0.3M sodium citrate in ddH2O pH 5.3; Sigma-Aldrich)) at 72oC for 5min and cells dehydrated (ethanol; 70%, 85%, 100% for 1min each). Cells were incubated with the denatured Vysis EWSR1 Break apart FISH Probe (3N5920, Vysis, Abbott Laboratories Ltd., UK) for 16h at 37oC, rinsed with post-hybridisation wash (0.3% nonident NP-40 (Merck Biosciences) in 2xSSC, pH 7.5) for 5min at room temperature, followed by two washes at 72oC for 2min and then one at room temperature for 1min. Cells were mounted in Faramount Mounting Medium (Dako, Agilent Technologies, UK) containing 4′,6-diamidino-2-phenylindole (0.2μg/ml, Sigma-Aldrich) to label nuclei and visualised using the Widefield Fluorescent Inverted Microscope Nikon Eclipse Ti-E (Nikon, UK).

*Reverse transcriptase polymerase chain reaction (RT-PCR).* RNA was extracted using the RNeasy Micro Kit (Qiagen, UK). Expression of the EWSR1-FLI1 /ERG mRNA was confirmed by RT-PCR, amplifying 50ng of RNA per sample. Total RNA was reverse transcribed using Superscript^TM^ III Reverse Transcriptase (Life Technologies, Thermo Fisher Scientific) [Roundhill, E.A. and S.A. Burchill, British journal of cancer, 2012. 106(6): p. 1224-33] and cDNA amplified using sequence specific reverse and forward primers and AmpliTaq Gold DNA polymerase (Invitrogen, Thermo Fisher Scientific) as previously described [Roberts, P., et al. Genes Chromosomes Cancer, 2008. 47(3): p. 207-20.]. PCR products were separated by 2% agarose (Sigma-Aldrich) gel electrophoresis and visualised, after staining with ethidium bromide (0.5μg/ml; Sigma-Aldrich), under UV light.

*Preparation of cytospins for immunocytochemistry (ICC).* Cytospins of ES and ES-CSCs were prepared by resuspension in PBS and centrifugation onto Superfrost plus glass slides (Thermo Fisher) at 1000g for 3 min using a ROTORFIX 32A centrifuge (Hettich, Tuttlingen, Germany). Excess PBS was removed by aspiration and slides centrifuged at 3000g for a further 1 min.

*Immunocytochemistry for CD99.* Cells were fixed in 4% paraformaldehyde (Sigma-Aldrich) in PBS (Sigma-Aldrich) for 30min at room temperature. CD99 protein expression was detected using the mouse EnVision+ System-HRP (DAB) kit (Dako, Agilent Technologies) and the CD99 mouse monoclonal antibody (1:50 for 1h at room temperature, M3601, clone 12E7 (Dako)) or corresponding mouse isotype control (4μg/ml, Negative Control Mouse IgG1, X0931 (Dako)). Cells were visualised using the Liquid DAB Substrate Chromogen System for peroxidase (Dako), counter-stained with 0.1% Mayer’s haematoxylin at room temperature for 15s. The ES cell line, TC-32, was included as a positive control for CD99 expression. Cells were visualised by light microscopy (Zeiss Axioplan microscope; Zeiss, UK). Protein expression of each target was scored manually by two independent reviewers.

*Western blotting.* Equal protein (25μg) loading was confirmed by blotting for β-actin (0.4μg/ml, A5441, Sigma-Aldrich). Membranes were probed for CD99 (0.1μg/ml, MIC2 (1C3), SC-53898, Santa Cruz Biotechnology Inc., USA) and neurexin-1 (2μg/ml, Anti-pan Neurexin-1, ABN161-I, Millipore) overnight at 4oC and expression detected using goat anti-mouse (1:5000, 170-6516 (Bio-Rad, UK) and goat anti-rabbit (1:5000, 4010-05 (Southern Biotech, Alabama, USA) secondary antibodies for 1h at room temperature. Protein bands were visualised and quantified using GelDoc Imaging System (Bio-Rad) using Luminata^TM^ Forte Western HRP Substrate (Millipore, UK).
