# Supplementary material for: RNA sequencing and functional studies of patient-derived cells reveal that neurexin-1 and regulators of this pathway are associated with poor outcomes in Ewing sarcoma
Source: Cell Oncol (Dordr). 2021 Aug 17;44(5):1065–85. doi: 10.1007/s13402-021-00619-8 (PMC8516792; doi:10.1007/s13402-021-00619-8)
Supplement: Supplementary file 5 — (DOCX 13 kb) [file 13402_2021_619_MOESM5_ESM.docx]

**Additional file 4. Method to evaluate the prognostic potential of differentially expressed proteins.**

*Preparation of tumour sections for immunohistochemistry (IHC).* Protein expression was detected on paraffin embedded (5μm; deparaffinisation in xylene and decreasing concentrations of ethanol, antigen retrieval in boiling 10mM citric acid in ddH2O (ph6) for 12min; Supplementary Data 3) and frozen tumour sections (5μm).

*Immunohistochemistry (IHC).* Tumour sections were fixed and permeabilised (Supplementary Data 3) and protein expression was detected using the species appropriate EnVision+ System-HRP (DAB) kit (Dako) and target specific (Additional Data 3) or corresponding isotype control antibodies (4μg/ml, Negative Control Mouse IgG1, X0931 (Dako) and 20μg/μl, Normal Rabbit Serum Control Ig mix, 086199 (Life Technologies)). Optimum antibody concentrations were determined empirically using positive control tissues (Additional Data 3). Cells were visualised using the Liquid DAB Substrate Chromogen System for peroxidase (Dako), counter-stained with 0.1% Mayer’s haematoxylin at room temperature for 15s. Cells were visualised by light microscopy (Zeiss Axioplan microscope). Protein expression of each target was scored manually by two independent reviewers. All samples were scored using the H score [Detre, S., G. Saclani Jotti, and M. Dowsett, J Clin Pathol, 1995. 48(9): p. 876-8.] taking into account the number of positive cells and intensity of expression (low (+), medium (++) and high (+++)).
