# Supplementary material for: RNA sequencing and functional studies of patient-derived cells reveal that neurexin-1 and regulators of this pathway are associated with poor outcomes in Ewing sarcoma
Source: Cell Oncol (Dordr). 2021 Aug 17;44(5):1065–85. doi: 10.1007/s13402-021-00619-8 (PMC8516792; doi:10.1007/s13402-021-00619-8)
Supplement: Supplementary file 7 — (PDF 419 kb) [file 13402_2021_619_MOESM7_ESM.pdf]

Additional file 6 Table S3

Full or alternative gene name	Gene	MSC, ESC or CSC associated	Mean read count (±s.d.) determined by total RNA sequencing	GSE17618	
				EFS	OS
Activated leukocyte cell adhesion molecule, CD166	ALCAM	MSC	19205 ±5938	NS	NS
Caveolin 1	CAV1	MSC	30965 ±14419	NS	NS
Leukocyte antigen MIC3	CD9	ESC	1231 ±1010	NS	NS
Stro-1	CD34	MSC	32 ±23	NS	NS
Epican	CD44	MSC	28295 ±9041	NS	NS
DNA (cytosine-5)-methyltransferase 3B	DNMT3B	ESC	184 ±69	NS	NS
Endoglin	ENG	MSC	4811 ±4219	NS	NS
Gamma-amiobutyric acid receptor subunit beta-3	GABRB3	ESC	144 ±144	NS	NS
Intracellualr adhesion molecule 1, CD54	ICAM1	MSC	1010 ±789	NS	NS
Integrin beta-1	ITGB1	MSC	116046 ±23980	NS	NS
Mast/stem cell growth factor receptor Kit	KIT	MSC	380 ±363	KM p=0.012, HR=2.37 p=0.015	KM p=0.039, HR=2.21 p=0.044
Podocalyxin	PODXL	ESC	2469 ±2216	NS	NS
Octamer-binding protein 4, OCT-4	POU5F1	MSC, ESC, pluripotency	20 ±15	NS	NS
Thy-1 membrane glycoprotein	THY1	ESC, MSC	11871 ±5526	NS	NS
Vimentin	VIM	MSC	70098 ±11898	NS	NS
Aldehyde dehydrogenase E3 isozyme	ALDH	CSC	16±23	NS	NS
Prominin-1	CD133	CSC	1±4	KM p=0.00096, HR=3.32 p=0.002	KM p=0.0026, HR=3.57 p=0.005
P-glycoprotein, MDR1 protein, ABCB1	Pgp	CSC	21±58	NS	NS
Multi-drug resistance associated protein 1, MRP1, ABCC1	ABCC1	CSC	6081±2266	KM p=0.0023, HR=3.06 p=0.003	KM p=0.0055, HR=3.23 p=0.009
